# Combined Effects of Soil Silicon and Host Plant Resistance on Planthoppers, Blast and Bacterial Blight in Tropical Rice

**DOI:** 10.3390/insects13070604

**Published:** 2022-07-01

**Authors:** Quynh Vu, Gerbert Sylvestre Dossa, Enrique A. Mundaca, Josef Settele, Eduardo Crisol-Martínez, Finbarr G. Horgan

**Affiliations:** 1 Cuulong Delta Rice Research Institute, Tan Thanh, Thoi Lai District, Can Tho 905660, Vietnam; vquynh@gmail.com; 2 Helmholtz Centre for Environmental Research, Theodor-Lieser-Strasse 4, 06120 Halle, Germany; josef.settele@ufz.de; 3 International Rice Research Institute, Makati 1226, Philippines; g.dossa@hotmail.com; 4 Escuela de Agronomía, Facultad de Ciencias Agrarias y Forestales, Universidad Católica del Maule, Casilla 7-D, Curicó 3349001, Chile; emundaca@ucm.cl (E.A.M.); eduardocrisol@gmail.com (E.C.-M.); 5 German Centre for Integrative Biodiversity Research, Puschstrasse 4, 04103 Leipzig, Germany; 6 Institute of Biological Sciences, University of the Philippines (UPLB), Los Baños 4031, Philippines; 7 EcoLaVerna Integral Restoration Ecology, Bridestown, Kildinan, T56 P499 County Cork, Ireland; 8 Association of Fruit and Vegetable Growers of Almeria (COEXPHAL), Carretera de Ronda 11, 04004 Almeria, Spain; 9 Centre for Pesticide Suicide Prevention, Centre for Cardiovascular Science, University of Edinburgh, Edinburgh EH16 4TJ, UK

**Keywords:** agroecology, brown planthopper, fertilizer, host-plant resistance, integrated pest management, *Magnaporthe grisea*, near-isogenic lines, rice seedlings, whitebacked planthopper, *Xanthomonas oryzae* pv. *oryzae*

## Abstract

**Simple Summary:**

Rice is often attacked by several herbivores and plant pathogens at the same time. Public research has mainly focused on enhancing rice resistance against these biotic stresses by selecting rice lines with resistance genes during breeding programs. However, rice resistance to biotic stresses is also affected by soil nutrients, including available nitrogen and silicon. Nitrogen tends to reduce resistance, but silicon can increase resistance. We assessed the effects of combining soil silicon with host plant resistance against rice planthoppers, blast disease, and bacterial blight disease. We used pure silicon (SiO_2_) to avoid the confounding effects of nutrients associated with silicates. We also assessed the effects of nitrogenous fertilizer on silicon-augmented resistance to planthoppers. We found that high nitrogen diminishes the capacity of soil silicon and host resistance to reduce planthopper fitness (i.e., nitrogen was antagonistic); but that silicon counters nitrogen-related reductions in rice antixenosis defenses (e.g., repellency) against gravid female planthoppers (i.e., an additive effect of silicon and resistance). Silicon augmented resistance against blast and bacterial blight, but the effects were most apparent on susceptible varieties. Plants infected with bacterial blight generally grew larger in silicon amended soils. We discuss how silicon improves seedling quality by augmenting broad-spectrum resistance.

**Abstract:**

Soil silicon enhances rice defenses against a range of biotic stresses. However, the magnitude of these effects can depend on the nature of the rice variety. We conducted a series of greenhouse experiments to examine the effects of silicon on planthoppers (*Nilaparvata lugens* [BPH] and *Sogatella furcifera* [WBPH]), a leafhopper (*Nephotettix virescens* [GLH]), blast disease (*Magnaporthe grisea*) and bacterial blight (*Xanthomonas oryzae*) in susceptible and resistant rice. We added powdered silica gel (SiO_2_) to paddy soil at equivalent to 0.25, 1.0, and 4.0 t ha^−1^. Added silicon reduced BPH nymph settling, but the effect was negligible under high nitrogen. In a choice experiment, BPH egg-laying was lower than untreated controls under all silicon treatments regardless of nitrogen or variety, whereas, in a no-choice experiment, silicon reduced egg-laying on the susceptible but not the resistant (*BPH32* gene) variety. Stronger effects in choice experiments suggest that silicon mainly enhanced antixenosis defenses. We found no effects of silicon on WBPH or GLH. Silicon reduced blast damage to susceptible and resistant (*Piz*, *Piz-5* and *Pi9* genes) rice. Silicon reduced damage from a virulent strain of bacterial blight but had little effect on a less virulent strain in susceptible and resistant (*Xa4*, *Xa7* and *Xa4* + *Xa7* genes) varieties. When combined with resistance, silicon had an additive effect in reducing biomass losses to plants infested with bacterial blight (resistance up to 50%; silicon 20%). We discuss how silicon-containing soil amendments can be combined with host resistance to reduce biotic stresses in rice.

## 1. Introduction

Silicon is the most abundant element on the Earth’s crust [1]. Consequently, silicon-rich compounds are frequent biproducts and wastes of industrial processes that involve combustion or mining [2,3]. Furthermore, silicon is accumulated in plants as they grow and develop such that composts derived from decaying plant materials are often relatively rich in silicon [4]. Silicon contributes to the mechanical structure of plants and has been associated with plant resistance to a range of pests and diseases [5,6,7]. This is at least partly due to silicon deposits in plant tissues that impede mechanical damage to the plants by herbivores, particularly leaf-chewers and stemborers [5,6]. However, there is now considerable evidence to suggest that silicon in plants is also involved in defense signaling pathways and, therefore, enhances induced plant defenses against pathogens and pests [8,9,10]. These induced defenses may include silicon-related changes to induced volatiles that draw in the parasitoids or predators of herbivores [11,12], thereby further protecting the plant.

Rice will often accumulate relatively high concentrations of silicon in leaves and shoots [13]. However, the final silicon content of rice plants depends on several factors, including plant age, the variety’s capacity to uptake and distribute silicon around the plant, as well as the content of silicon available in the soil [14,15,16]. Soil silicon content varies regionally and is affected by crop management practices, particularly by residue management after harvest [17,18]. Soils that are relatively deficient in silicon can be treated with a range of amendments to augment soil and plant silicon contents and to increase yields [5,6]. Certain additives, such as imidazole, can further enhance the uptake of silicon by plants [19,20]. Because many soil silicon amendments are relatively cheap or are widely available, adding silicon has been recommended as a viable means of improving soil quality while, at the same time, enhancing crop defenses against pests and pathogens [2,21,22,23].

Rice has become a model for much recent research on silicon-associated defenses against pests and diseases [1,23]. This is partly because of the importance of rice as the main staple for over 50% of the world’s population and because rice is attacked by a range of insect herbivores and plant pathogens [24,25]. A large number of studies have shown that soil amendments that include high levels of silicon (i.e., 15–70%) can directly increase rice yields while reducing damage from planthoppers (Delphacidae, Hemiptera), stemborers (Crambidae, Lepidoptera), leaffolders (Crambidae, Lepidoptera) and other pests [11,26,27,28,29,30,31]. Silicon soil amendments have also been shown to reduce the incidences of a range of rice diseases, including brown spot (*Bipolaris oryzae*, *Cochliobolus miyabeanus*), sheath blight (*Thanatephorus cucumeris*, *Rhizoctonia solani*), stem rot (*Magnaporthe salvinii*), and leaf scald (*Monographella albescens*) [21,32,33]. Research has also shown that soil silicon can reduce pest and disease damage in an additive manner when combined with biocontrol agents or pesticides [11,21]. However, despite considerable interest in developing rice varieties with resistance to herbivores and diseases [34,35,36,37,38] and a growing awareness of the role of silicon in constitutive and induced plant defenses [5,6], few studies have examined the interactions between silicon and host plant resistance, particularly for insect herbivores. A small number of studies have examined the effects of silicon amendments on the fitness and damage caused by stemborers, leaffolders, or stink bugs on susceptible and moderately resistant rice [28,39,40]. However, rice resistance to these pests is largely related to plant anatomy [41] and is not associated with any major resistance genes.

Unlike the case of stemborers and leaffolders, resistance against planthoppers and leafhoppers and against several rice diseases has been achieved through the marker-assisted selection of rice genotypes with major resistance genes [34,36,37,38,42]. Resistance against planthoppers has been attributed to several mechanisms, including antifeedants, antidigestants, repellant volatiles, and surface waxes that directly reduce planthopper fitness (i.e., feeding capacity, growth rates, egg laying, etc.). Several resistance genes are associated with induced defenses that are mediated through the Jasmonic Acid (JA) or Salicylic Acid (SA) pathways [42]. Resistance against rice diseases often involves induced gene-for-gene responses that recognize and target specific pathotypes [36,37,38]. Silicon soil amendments are thought to generally increase the efficacy of resistant rice (e.g., further reducing feeding capacity, growth rates or fecundity in herbivores, or reducing disease severity in pathogens) by enhancing defense signaling pathways [5,6,7]. Silicon might also counter the negative effects of nitrogenous fertilizers that tend to reduce host resistance against several insect herbivores [43], and particularly against planthoppers [44,45], by countering nitrogen-related softening of growing tissues (i.e., increasing the carbon: nitrogen [C:N] ratio of plant tissues).

In the present study, we examine the effects of silicon-based soil amendments on rice resistance to two planthoppers (the brown planthopper, *Nilaparvata lugens* (Stål) [BPH] and the whitebacked planthopper, *Sogatella furcifera* Horváth [WBPH]), a leafhopper (*Nephotettix virescens* (Distant) [Cicadellidae: Hemiptera] [GLH]) and two rice diseases: blast (*Magnaporthe grisea* (Herbert) Barr. [BL]) and bacterial blight (*Xanthomonas oryzae* pv. *oryzae* [Xoo]). BPH and WBPH are pests of intensified irrigated rice [34,45], and blast and bacterial blight are considered among the most serious diseases of rice in tropical Asia [36,46]. The genetic mechanisms underlying rice resistance to these organisms have been relatively well researched [34,36,37,38,42]. We designed our experiments with planthoppers to examine whether silicon amendments might counter the negative effects of nitrogenous fertilizers and assessed whether silicon affects rice antibiosis defenses, antixenosis defenses, or both. We also conducted experiments with BPH on susceptible varieties in the Philippines and in Vietnam because of different silicon levels in soils from the two regions (soils of the Mekong Region of Vietnam generally have low amounts of weatherable silicate materials compared to soils in the Philippines [14]). In our experiments with rice blast and bacterial blight, we assessed the effects of increasing soil silicon concentrations on disease progression using rice differentials (blast) and recently developed near-isogenic rice lines with relatively high and low virulent disease (bacterial blight) isolates. We predicted that silicon would play a greater role in resistance against the more virulent strain because of generally weak induced defenses against the isolate. To our knowledge, this is the first study to examine the combined effects of silicon, nitrogen and host resistance against rice planthoppers and the first to compare effects on low and high virulent disease isolates. We discuss the potential for including soil silicon amendments as a general approach in integrated rice pest and disease management.

## 2. Materials and Methods

### 2.1. Insect Herbivores

Experiments in the Philippines were conducted with BPH, WBPH, and GLH taken from colonies maintained at the International Rice Research Institute (IRRI). The GLH colony was initiated in 2008 with >500 individuals captured in rice fields at Laguna, Philippines. The BPH and WBPH colonies were initiated in 2009 using >500 individual planthoppers of each species collected from the same rice fields as GLH. We also conducted a number of bioassays with BPH from a colony maintained at the Southern Regional Plant Protection Centre (SRPPC) in Tien Giang Province, Vietnam. The colony was initiated in 2012 with individuals collected from rice fields at Ling Dinh. The planthoppers and leafhoppers were reared separately in aluminum mesh cages (91.5 × 56.5 × 56.5 cm (H × L × W)) under ambient light and temperature conditions in greenhouses at IRRI and SRPPC. All four colonies were maintained on the susceptible rice variety TN1 at >30 days after sowing (DAS). Rice plants were changed every week. All herbivore colonies used in the experiments were free of associated plant viruses (e.g., tungro, rice ragged stunt virus [RRSV], and rice grassy stunt virus [RGSV]) and parasitoids. BPH from Laguna was virulent against *Bph1*, *bph2*, *bph5*, *bph7*, *bph8*, *BPH18*, *BPH25,* and *BPH26* at the time of colony initiation, and WBPH was virulent against *Wbph2*, *WbphM1* and *WbphM2* [47]. BPH from Ling Ding had relatively high virulence against several resistance genes, including *Bph1*, *bph2*, *Bph3*, *bph4*, *bph8*, *bph9*, *BPH10,* and *BPH18* [47]. We conducted only limited experiments with GLH and present related results only in the Supplementary Information.

### 2.2. Disease Organisms

We used the M39-1-3-8-1 isolate in all bioassays with blast disease. We used two Philippines Xoo strains in our experiment with bacterial blight. These were PXO99 (activator of *Xa21*-mediated immunity [*avrXa21*]) and PXO145 (*avrXa4* + *avrXa7*). PXO145 was targeted by the *Xa4* and *Xa7* genes carried by the NILs used in the experiment, whereas PXO99 was expected to be largely virulent against the NILs and the susceptible checks. All isolates were provided by the plant pathology group at IRRI.

### 2.3. Plant Materials

In the Philippines, we used IR62 as a variety with relatively strong resistance to BPH, WBPH, and GLH [48]. Resistance in IR62 is thought to be derived from the highly resistant Indian landrace PTB33. Breeding experiments and bioassays with planthopper biotypes indicate that the variety contains the *BPH3* resistance locus, which includes the *BPH32* resistance gene [48,49]. We used IR22 as a susceptible variety. This variety has been shown to enhance the fitness of BPH (resulting in highly fecund females [50]) and was used as an alternative to the susceptible natal host TN1, such that all herbivores could be changed to a new variety (either susceptible or resistant) during the experiments. In Vietnam, experiments with BPH were conducted using the variety IR50404. This variety carries the *bph2* gene and has moderate resistance to BPH [51,52].

The experiments with blast were conducted using seven rice varieties. Varieties were selected based on their reactions to blast. We used three differential lines with known resistance to several blast isolates from the Philippines: IRBL9-W carries the *Pi9 (t)* gene; IRBLz5-Ca carries the *Piz-5* (=*Pi2*) gene, and IRBLz-Fu carries the *Piz* gene for blast resistance [36]. We used the highly susceptible varieties CO39 (for which the *M. grisea AVRI-CO39* locus specifies avirulence [53]) and Liangjiangxintuanheigu (LTH) [38], and the varieties IR22 and IR24 (*Pi20*) that have limited resistance to some blast isolates [54].

Five varieties were used in experiments with bacterial blight. These included IR24 as a susceptible check [35] and three near-isogenic lines carrying bacterial blight (Xoo) resistance genes. Near-isogenic lines were developed at IRRI using IR24 as the recurrent parent. The IRBB4 NIL contains the *Xa4* gene, the IRBB7 NIL contains the *Xa7* gene, and the IRBB67 NIL is a pyramided line possessing both genes (*Xa4* + *Xa7*) [35]. We included IR22 as a standard ‘susceptible’ variety in all the bioassays that we conducted in the Philippines (BPH, WBPH, GLH, BL, and Xoo).

All experiments were conducted under greenhouse conditions at ambient light and temperature (25–37 °C, L12:D12 at IRRI; 26–38 °C, L12:D12 at SRPPC). Plants were grown in pots (indicated below) and were tended daily, receiving water and weeded as necessary. The plants received no pesticide treatments, and, unless indicated, they received no added fertilizer.

### 2.4. Soil Preparation

Paddy soil was initially filled into plastic basins and mixed with different amounts of ground (powdered) silicon (SiO_2_). Pure silica (gel) was used to avoid the confounding effects of other elements in some silicon-based soil amendments [18]. The amounts of silica gel (SiO_2_) added to the pots were calculated on the basis of equivalents to field soils. For conversion from unit t ha^−1^ to g kg^−1^, we assumed a soil density of 1.5 g cm^−3^ and a depth of the topsoil horizon of 15 cm. Therefore, we applied 0.11, 0.44, and 1.78 g SiO_2_ Kg^−1^ as equivalents for 0.25, 1.0, and 4.0 t ha^−1^, respectively. We used a maximum of 4 t ha^−1^ because higher levels would be uneconomical at large field scales [23]. The SiO_2_-mixed soil was flooded for 14 days to allow the silicon to dissolve completely before seed sowing. After 14 days, the treated soils were used to fill pots. Potted plants in the Philippines for use in experiments with BPH, WBPH, and GLH received one of two nitrogen fertilizer regimes as zero-added fertilizer and nitrogenous fertilizer added at equivalent to 150 kg ha^−1^. Otherwise, soils received no added fertilizer. Fertilizer was applied as a basal treatment (50%) before sowing or transplanting and again at 5 days prior to the bioassays (50%).

### 2.5. Effects of Nitrogen and Silicon on Seedling Growth

We grew IR22 and IR62 plants in soil treated with each of the four silicon treatments and two nitrogen levels as described above. Seed was initially sown to trays filled with saturated paddy soil from Laguna rice fields. At 5 DAS, the seeds were individually transplanted to size-0 pots (7 × 11 cm; H × D: 2 nitrogen levels × 4 silicon treatments × 6 replicates = 48 pots). The plants were allowed to grow and develop for 35 days. At 35 days after transplanting (DAT), plants were carefully pulled from the soil, and the roots washed under gently running water. The plants were placed individually in paper bags and dried at 60 °C for 7 days in a forced draught oven. After drying, the plants were weighed on a precision balance. Dried IR22 plants (zero nitrogen) were ground (all plant parts together) using a mortar and pestle, and soil silicon was estimated gravimetrically as crude silica (%) after acid digestion [55]. Because of the small size of the seedlings, plants were pooled within treatments for analysis. We focused on IR22 because this was a standard control variety in all our experiments in the Philippines (Table S1).

### 2.6. Effects of Soil Silicon on Rice Resistance to Planthoppers and Leafhoppers

#### 2.6.1. Antibiosis Tests

We conducted nymph survival tests with all three herbivore species in the Philippines but with BPH alone in Vietnam. For each species (BPH, WBPH, and GLH) and rice variety (IR22 and IR62 in the Philippines, IR50404 in Vietnam), the protocols were the same (Table S1). Rice seed was planted in pots of 7 × 11 cm (H × D) with treated soils at three seedlings per pot. The seedlings were allowed to develop for 20 days, after which the pots were each covered by an acetate cage (45 × 5 cm, H × D) with a mesh top. At 20 DAS, plants were infested with 10 neonate planthoppers or leafhoppers. The nymphs were allowed to grow and develop for 15 days, after which the insects were collected using a vacuum sampler, and the rice plants were destructively harvested. The rice plants and planthoppers were placed in separate paper bags and dried at 60 °C for 1 week. The planthoppers or leafhoppers were then counted and weighed, and the development stages noted. The dried rice plants were also weighed.

An adult survival bioassay was also conducted with BPH in Vietnam. Rice plants were seeded in 15 × 15 cm (H × D) pots with each of the soil treatments. After 25 days, the pots were individually covered with acetate cages (112.5 × 11.5 cm (H × D)). During the bioassay, 10 adult BPH (unmated females) were confined to pots with five rice plants (IR50404 at 30 DAS) under each of the four soil treatments (Table S1). The adult BPH were allowed to feed for up to 5 days, after which the seedlings were searched for surviving adults, and the numbers of survivors were recorded.

Oviposition bioassays were conducted with BPH and WBPH on IR22 and IR62 in the Philippines and with BPH on IR50404 in Vietnam (Table S1). For the experiments, rice plants were seeded in 15 × 15 cm (H × D) pots with each of the soil treatments. After 15 days, the pots were individually covered with acetate cages (112.5 × 11.5 cm (H × D)). At 20 DAS, each plant was infested with a pair (1 male, 1 female) of recently emerged adult planthoppers (either BPH or WBPH). The planthoppers were allowed to feed and oviposit on the plants for three days, after which the adults were removed. The plants were then carefully pulled from the soil and dissected under a stereomicroscope (×10 magnification) to locate egg batches. The number and location (relative to the leaf midrib) of egg batches were noted. Each egg batch was examined, and the numbers of eggs were recorded.

Experiments with each herbivore species were set up as randomized block designs in the Philippines and completely randomized designs in Vietnam, each with 6 replicates.

#### 2.6.2. Antixenosis Tests

We also conducted choice-settling bioassays with BPH, WBPH, and GLH nymphs on IR22 and IR62 in the Philippines, choice oviposition bioassays with IR22 and IR62 in the Philippines, and a choice oviposition bioassay with BPH on IR50404 in Vietnam.

In the Philippines, choice nymph settling and oviposition bioassays (Table S1) were conducted using arenas of 1 m^3^, each consisting of a wooden frame with mesh (0.2 mm) sides and a mesh top. Each arena had 16 rice plants (2 varieties × 4 silicon levels × 2 nitrogen levels) individually planted in size-0 pots (7 × 11 cm; H × D). At the time of the experiments, the plants were at 20 DAS. Plants were placed in the arenas in a 4 × 4 configuration, with treatments and varieties randomly assigned to positions for each replicate.

For the nymph settling experiment in the Philippines, 160 nymphs (≤2nd instar) were released to each arena (placing all nymphs on the arena floor at the center of the plants). Nymphs were allowed to interact with the plants for 5 days after which the numbers of nymphs on each plant were counted. The nymphs were then collected, and the plants were destructively harvested, placed in paper bags, dried (at 60 °C for 7 days), and weighed.

For the choice oviposition experiment in the Philippines, 32 mated, gravid females were placed on the arena floor at the center of the 16 plants. The females were allowed to interact with the plants and oviposit for 3 days, after which the females were removed, and the plants were destructively harvested by carefully pulling each plant from the soil and rinsing the roots under cold water. Each plant was examined under a stereomicroscope and dissected to find the egg clusters. The numbers of clusters and the numbers of eggs in each cluster were recorded. The plants were then dried (at 60 °C for 7 days) and weighed.

In Vietnam, the choice oviposition bioassays were conducted using plastic arenas of 50 × 50 × 70 cm (L × W × H). The arenas each had four rice plants (20 DAS) in size-0 pots (7 × 11 cm; H × D), with one pot for plants grown under each silicon treatment (including the zero-silicon control). Plants were arranged in a circular configuration taking care that the foliage did not touch other plants or the arena walls. A total of 10 mated BPH females were released to each arena at the center of the four potted plants. The adult females were allowed to interact with the plants and oviposit for 3 days, after which the plants and planthopper eggs were sampled as described above. All choice bioassays were replicated six times.

### 2.7. Effects of Soil Silicon on Rice Resistance to Blast

Plants (14 DAS) of seven rice genotypes described above were used to test the expression of blast disease under different levels of silicon application (Table S1). Plants were inoculated by spraying a solution diluted with specific blast isolate M39-1-3-8-1. In order to observe blast symptoms, the inoculated plants were incubated in a mist room at 26 °C (12D:12N). The plants were evaluated after 7 days of incubation using the Standard Evaluation System (SES) for scoring blast severity [56].

### 2.8. Effects of Soil Silicon on Rice Resistance to Two Strains of Bacterial Blight

Rice plants at 21 DAS and 45 DAS of five rice genotypes (as described above) were used to test Xoo expression under different levels of soil silicon (Table S1). The plants were all sown at the same time and were evaluated in sequence. Inoculums of the PXO99 and PXO145 isolates were prepared from 3-day-old cultures. Plants were inoculated by the leaf clipping method at 21 DAS (early inoculation) or 45 DAS (late inoculation) using scissors to cut the tips of the leaves after dipping into the corresponding isolate [35]. At 11 (early inoculation) or 14 (late inoculation) days after inoculation (DAI), plants were evaluated by measuring the length of disease lesions using digital calipers on the inoculated leaves. After lesions were measured, the plants were destructively sampled by carefully pulling the plants from the soil (keeping the roots intact). The roots were washed under gently running water, and the plants placed in paper bags and dried in a forced draught oven at 60 °C for 7 days. After drying, the plants were weighted on a precision balance to estimate dry weight.

### 2.9. Data Analyses

Plant growth rate and herbivore no-choice experiments conducted with BPH, WBPH, and GLH in the Philippines were analyzed separately using univariate general linear models (GLM) with plant dry weight, nymph survival (arcsine transformed), development to adults (arcsine transformed), nymph weight, the number of eggs per plant and plant weight loss as dependent variables and rice variety, nitrogen level and silicon level as independent variables. Block effects and the covariate plant weight (for herbivore experiments) were initially included in the analyses but were removed where they had no effect. The effects of silicon were assessed using Tukey post hoc tests and by examining polynomial contrasts. For analysis of the no-choice experiments conducted with BPH in Vietnam, univariate GLM examined the effects of silicon only and tested for polynomial contrasts. For the Vietnamese data, plant weight was initially included as a covariate.

Results from the choice nymph settling and egg-laying experiments (proportion of nymphs settled, proportion of eggs on plants) were ranked within experimental units (i.e., individual arenas) and analyzed using univariate GLM with arenas included as blocks. For experiments with blast, univariate GLMs included rice genotypes and silicon levels as main factors. We conducted repeated measures GLMs with the results (lesion length, plant weight) from the experiment with Xoo. Plant stage (21 and 45 DAS) represented the repeated measure, and the analysis included rice genotype, bacterial isolate, and silicon as main factors. For both experiments with plant diseases, we used Tukey tests to identify homogenous silicon groups. Residuals were plotted to verify data homogeneity and normality after all parametric analyses. All analyses were conducted using IBM SPSS Statistics (version 21) (New York, NY, USA).

## 3. Results

### 3.1. Seedling Growth under Varying Soil Silicon

**Figure 1:**
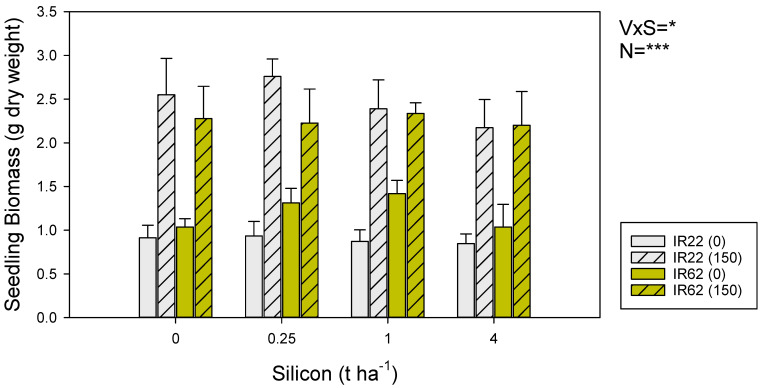
Biomass (g dry weight) of IR22 (gray bars) and IR62 (yellow bars) seedlings at 35 DAT. Seedlings were grown in pots with zero added nitrogen (solid bars) and nitrogen equivalent to 150 kg ha^−1^ (hatched bars). Pots received one of four silicon treatments. Standard errors are shown (sample size = 6). Summary statistics for univariate general linear model are indicated as V *×* S = variety × silicon level interaction, N = nitrogen, * = *p* ≤ 0.05, and *** = *p* ≤ 0.005.

IR22 and IR62 plants attained a similar biomass by 35 DAS (F_1,80_ = 0.158, *p* = 0.692). Nitrogen increased the biomass of both varieties (F_1,80_ = 105.556, *p* < 0.001). Silicon tended to increase seedling biomass of IR62 seedlings at 0.25 and 1 t ha^−1^, but the same effect was not apparent with IR22 seedlings (F_1,80_ = 4.058, *p* = 0.047) (Figure 1). All other interactions were non significant. The estimated crude silica content of IR22 seedlings were 1.5% (controls), 1.5% (0.25 t SiO_2_ ha^−1^), 2.0% (1.0 t ha^−1^) and 2.2% (4.0 t ha^−1^).

### 3.2. Effects of Resistance, Nitrogen and Silicon on Antibiosis Defenses against Planthoppers and Leafhoppers (Philippines)

**Figure 2:**
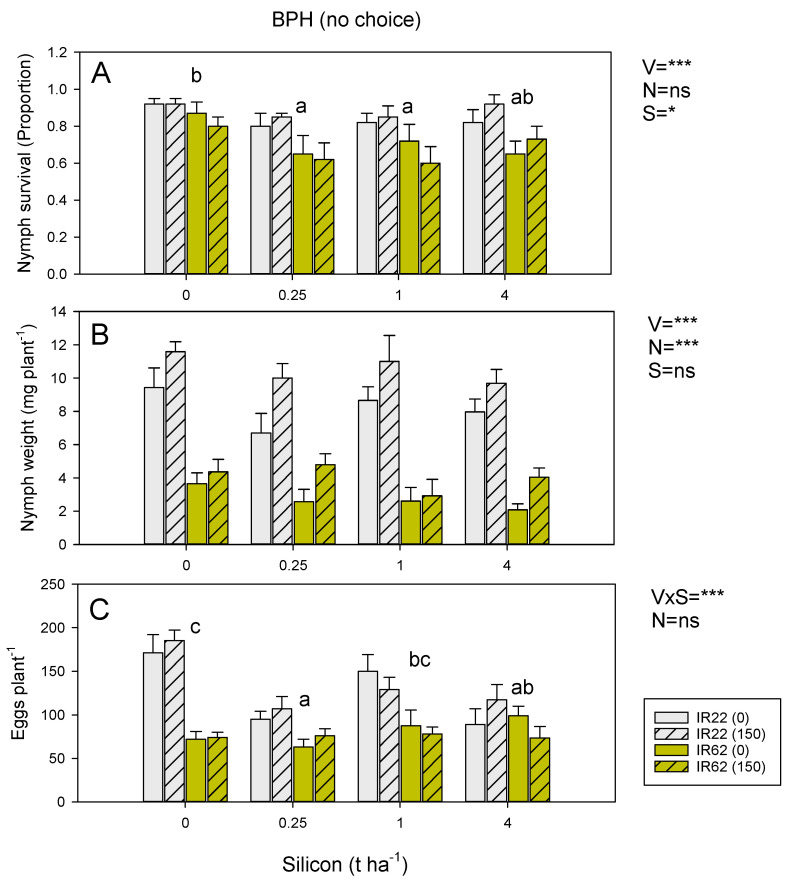
Effects of host resistance, soil nitrogen and soil silicon on (**A**) BPH nymph survival, (**B**) BPH nymph weight gain, and (**C**) BPH egg laying during no-choice experiments conducted in the Philippines. The experiments were conducted using IR22 (susceptible = gray bars) and IR62 (resistant = yellow bars) at 0-added (solid bars) and the equivalent of 150 Kg ha^−1^ (hatched bars) added nitrogen. Soil was amended with 0, 0.25, 1 and 4 t ha^−1^ equivalents of silicon. Summary statistics for univariate general linear models are indicated as V = variety, N = nitrogen level, S = silicon, V × S = variety × silicon interaction, ns = *p* > 0.05, * = *p* ≤ 0.05, and *** = *p* ≤ 0.001. Standard errors are indicated (sample size = 6); lowercase letters indicate homogenous silicon groups. See also Table S2.

BPH nymphs had lower survival and lower weight gains on IR62 plants (Figure 2A,B), and fewer eggs were laid on IR62 (Figure 2C). Nitrogen accelerated nymph development (Table S2) and promoted nymph weight gain (Figure 2B). Nitrogen had no effect on egg laying per plant (Figure 2C), but the number of eggs per plant dry weight was reduced in the larger nitrogen-treated plants (Table S2). Silicon amendments reduced nymph survival, with the largest declines at low to intermediate silicon levels (0.25 and 1 t ha^−1^: Figure 2A). Silicon amendments resulted in a decline in egg laying on IR22 but not on IR62; the largest effect on egg laying was apparent at 0.25 t ha^−1^ of silicon (Figure 2C). WBPH nymph survival and weight gain were lower on IR62 than on IR22 (*p* < 0.001) and were lower in soil without added nitrogen (*p* < 0.05: Table S3). There was a significant variety × nitrogen interaction (*p* < 0.01) because of higher nymph weights on IR22 under added nitrogen but similar weights of nymphs on IR62 at both nitrogen levels (Table S3). WBPH laid fewer eggs on IR62 seedlings (*p* < 0.001: Table S3), and the number of eggs per plant weight was lower on nitrogen-treated plants (Table S3). There was no effect of silicon amendments on nymph survival, nymph weight gain, or egg laying, and no significant interactions (Table S3). Similarly, GLH nymph survival and weight gain were lower on IR62 than on IR22 and lowered under zero nitrogen (Table S4). However, there was no apparent effect of adding soil silicon (Table S4).

### 3.3. Effects of Resistance, Nitrogen and Silicon on Antixenotic Defenses against Planthoppers and Leafhoppers (Philippines)

**Figure 3:**
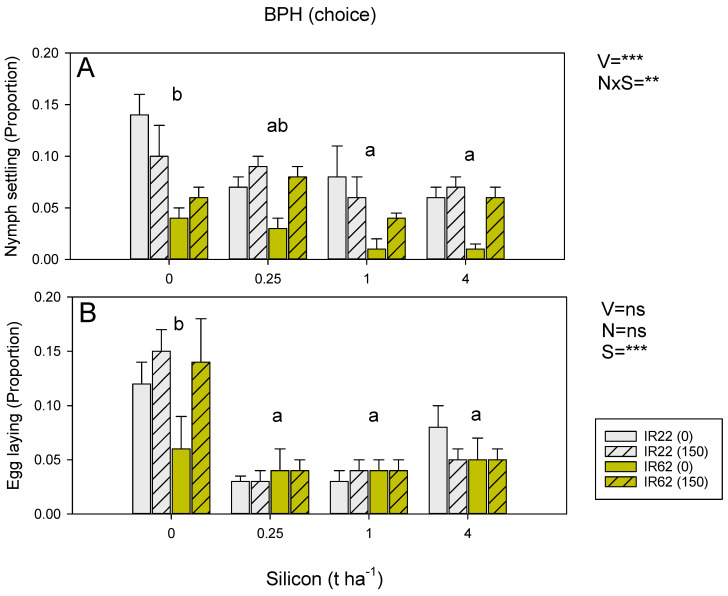
Effects of host resistance, soil nitrogen and soil silicon on (**A**) BPH nymph settling, and (**B**) BPH egg laying during choice experiments conducted in the Philippines. The experiments were conducted using IR22 (susceptible = gray bars) and IR62 (resistant = yellow bars) at 0-added (solid bars) and the equivalent of 150 Kg ha^−1^ (hatched bars) added nitrogen. Soil was amended with 0, 0.25, 1 and 4 t ha^−1^ equivalents of silicon. Standard errors are indicated (sample size = 6). Summary statistics for univariate general linear models are indicated as V = variety, N = nitrogen level, S = silicon, N × S = nitrogen × silicon interaction, ns = *p* > 0.05; ** = *p* ≤ 0.01, and *** = *p* ≤ 0.001; lowercase letters indicate homogenous silicon groups. See also Table S5.

In the choice experiment, more BPH nymphs settled on IR22 plants and plants under high nitrogen (Figure 3A). Soil silicon reduced settling (linear contrast *p* < 0.001: Figure 3A); however, there was a significant silicon × nitrogen interaction because the effects of silicon were more apparent for plants without added nitrogen. Settling preferences and consequent feeding resulted in variety, nitrogen, and silicon effects on final plant weight; however, the three-way interaction was also significant (Table S5). There was no apparent effect of soil silicon on settling by WBPH nymphs (Table S6) or GLH nymphs (Table S4). Both WBPH and GLH settled less on IR62 plants, and fewer WBPH nymphs settled on low-nitrogen plants compared to high-nitrogen plants (Tables S4 and S6).

There was no effect of variety or nitrogen on BPH egg laying in the choice experiment (Figure 3B). Silicon strongly affected egg-laying, with females avoiding plants treated with each of the three silicon levels (Figure 3B). Adult WBPH laid fewer eggs on IR62 plants and on low nitrogen plants in the choice experiments, but silicon had no apparent effects (Table S6).

### 3.4. Effects of Silicon on Antibiosis and Antixenosis Defenses against BPH (Vietnam)

**Figure 4:**
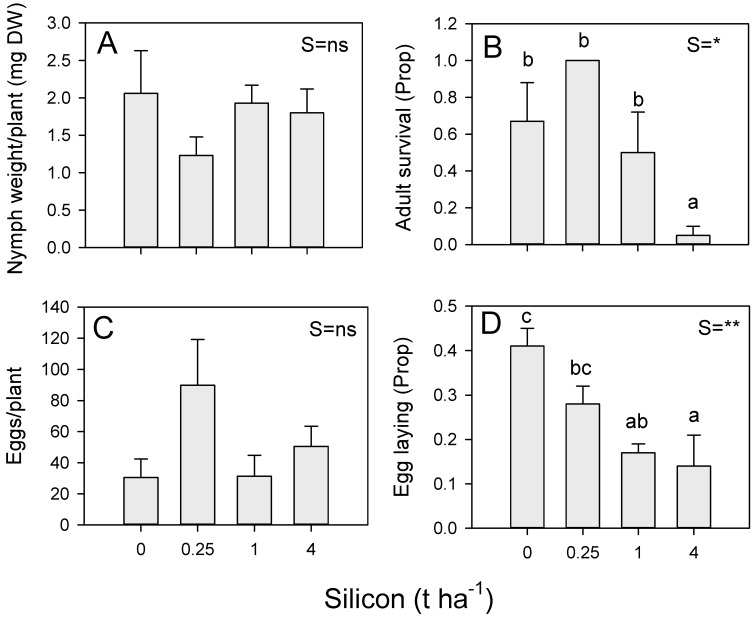
Effects of soil silicon on BPH (**A**) nymph weights, (**B**) adult survival and (**C**) egg laying during no-choice experiments conducted using rice variety IR50404 in Vietnam, and on BPH (**D**) egg laying during a corresponding choice experiment. Soil was amended with 0, 0.25, 1 and 4 t ha^−1^ equivalents of silicon. Standard errors are indicated (sample size = 6). Summary statistics for univariate general linear models are indicated as S = silicon, ns = *p* > 0.05, * = *p* ≤ 0.05 and ** = *p* ≤ 0.01; lowercase letters indicate homogenous silicon groups. See also Tables S7 and S8.

There was no effect of soil silicon on nymph survival and weight gain or on egg laying in the no-choice bioassays conducted in Vietnam (Figure 4; Table S7). Female survival during the oviposition experiments was affected by silicon, with a significant linear decline (*p* = 0.011) in survival with increasing soil silicon levels (Figure 4B; Table S7). Egg laying in the choice experiment declined linearly (contrast *p* < 0.001) with increasing soil silicon (Figure 4D; Table S7).

### 3.5. Effect of Resistance and Silicon on Blast Severity

**Figure 5:**
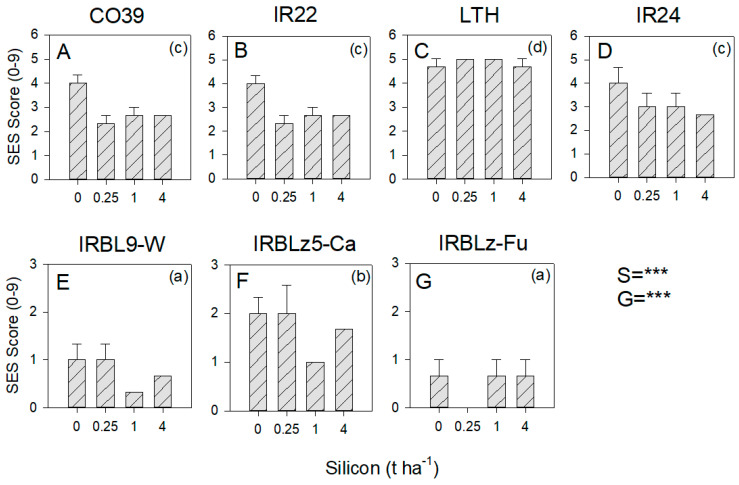
Effects of soil silicon on blast disease in 7 rice varieties: Varieties (**A**) CO39, (**B**) IR22, (**C**) LTH, (**D**) IR24 were susceptible to blast, whereas varieties (**E**) IRBL-W, (**F**) IRBLz5-Ca, and (**G**) IRBLz-Fu were resistant to blast. Blast incidence was evaluated using the Standard Evaluation System (SES) for leaf blast. Soil was amended with 0, 0.25, 1 and 4 t ha^−1^ equivalents of silicon. Standard errors are indicated (sample size = 3). Summary statistics for the univariate general linear model are indicated as S = silicon level interaction, G = rice genotype, and *** = *p* ≤ 0.005; lowercase letters in parentheses indicate homogenous variety groups; note differences in scales. See also Table S9.

Rice lines IRBL9-W, IRBLz-Fu, and IRBLz5-Ca were significantly more resistant to blast infection than the remaining four lines (F_3,56_ = 99.369, *p* < 0.001: Figure 5). Silicon produced a linear decrease in disease severity (F_3,56_ = 5.048, *p* < 0.001; contrast *p* < 0.001: Figure 5). There were no significant interactions (Table S8).

### 3.6. Effect of Resistance and Silicon on Xoo Severity

**Figure 6:**
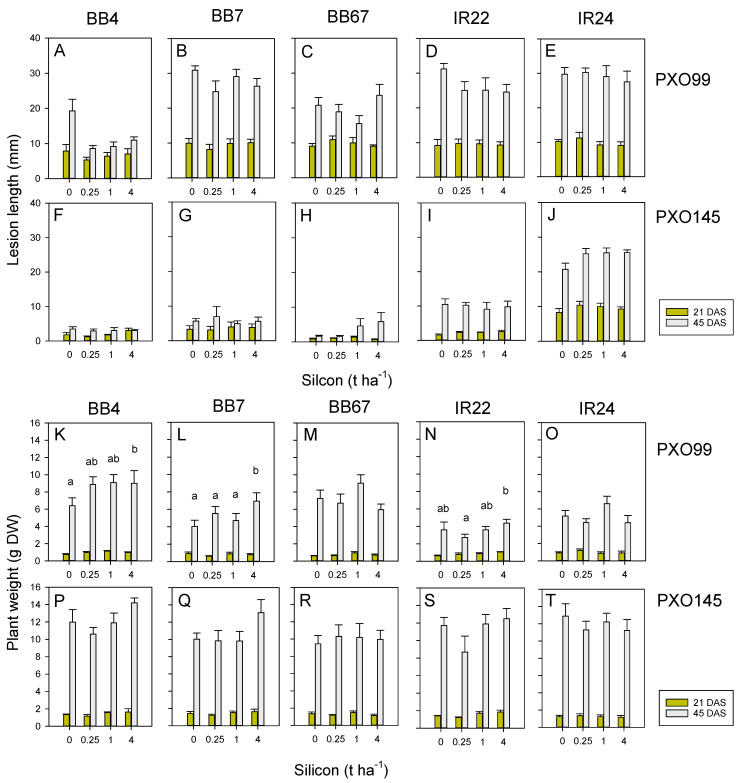
Effects of soil silicon on damage to rice from two strains of bacterial leaf blight (Xoo) to five rice varieties. Plants were challenged with (**A**–**E**,**K**–**O**) the highly virulent PXO99 strain and (**F**–**J**,**P**–**T**) the moderately virulent strain PX0145, under greenhouse conditions. The near-isogenic lines BB4 (**A**,**F**,**K**,**P**) and BB7 (**B**,**G**,**L**,**Q**) have monogenic resistance against PXO145 through the *Xa4* and *Xa7* R-genes, respectively. The pyramided line BB67 (**C**,**H**,**M**,**R**) contains both R-genes for resistance to PXO145. The varieties IR22 (**D**,**I**,**N**,**S**) and IR24 (**E**,**J**,**O**,**T**) were used as susceptible controls; however, IR22 was found to be also resistant to PXO145 (**I**). The effects of Xoo were evaluated as lesion length (**A**–**J**) and rice plant weight (**K**–**T**) with evaluations conducted at 32 (21 DAS + 11 DAI; yellow bars) and 59 (45 DAS + 14 DAI; grey bars) days. Soil was amended with 0, 0.25, 1 and 4 t ha^−1^ equivalents of silicon. Lowercase letters in (**K**–**T**) indicate homogenous silicon groups based on partitioned analyses of varieties. Standard errors are indicated (sample size = 6). See also Table S10, and see Table S11 for results of repeated-measure GLMs.

Xoo lesions increased over the course of the experiment (F_1,200_ = 747.102, *p* < 0.001), were larger where plants had been inoculated with PXO99 (F_1,200_ = 820.027, *p* < 0.001) and were significantly affected by rice genotype (IR24 > IR22 = BB7 > BB67 > BB4: F_4,200_ = 152.053, *p* < 0.001) (Figure 6; Table S10). There were several significant interactions between genotype and both plant age and bacterial blight strain, largely due to the varying progression rates of lesions as plants gained size (see within-subject effects in Table S11). There was a significant Xoo strain × genotype interaction because IR24 had no apparent resistance to either strain, but all other varieties were more resistant to PXO145 than PXO99 (F_4,200_ = 39.687, *p* < 0.001). There were significant silicon × Xoo strain (F_3,200_ = 6.014, *p* < 0.001) and silicon × genotype (F_4,200_ = 1.910, *p* = 0.035) interactions because silicon tended to reduce PXO99 lesions, but had no apparent effect on PXO145 lesions, and because of a lack of effect of silicon on lesions in IR24 (Figure 6; Tables S10 and S11).

The results based on plant weights were largely similar to those with lesion lengths; however, silicon had a significant effect on plant weight, with larger plants after treatments with 1 and 4 t ha^−1^ of silicon than 0.25 t ha^−1^ of silicon (F_3,200_ = 4.231, *p* = 0.006: Figure 6). Furthermore, BB4 plants were larger than all other varieties at the end of the experiment (F_4,200_ = 8.765, *p* < 0.001: Figure 6). There was a silicon × genotype interaction because of similar plant weights of BB67 and IR24 across all silicon levels, but improved weights of the remaining varieties at higher silicon levels (F_12,200_ = 2.005, *p* = 0.025). There was no Xoo strain × silicon interaction for plant weights (Table S11).

## 4. Discussion

### 4.1. Direct Effects of Soil Silicon on Rice Growth

A number of studies, using a range of different silicon sources and different rice varieties, have indicated that by increasing soil silicon content, the densities of silica crystals in rice leaves and the silicon content of rice plants, particularly the leaves and stems, increase [10,15,39,57,58,59]. Although our estimates of silica were rudimentary, the silica content of IR22 plants did increase with increasing concentrations of applied silica gel. Klotzbücher et al. (2018) [15] found that silicon levels in rice leaves without added soil silicon were between 1.5 and 2.5% of dry weight, rising to as much as 3.5% at 1.5 t SiO_2_ ha^−1^. We did not analyze any varieties other than IR22 in our experiments. The capacity to assimilate silicon differs between varieties, but this has not been consistently related to pest resistance or susceptibility where plants possess major resistance genes [39,40,60]. Soil amendments that contain high amounts of silicon have also been associated with increasing rice seedling weight [61,62,63], higher C:N ratios in leaf tissues [57,63], higher sugar and protein contents [57], and higher chlorophyll contents [64]. Ultimately, soil silicon amendments can lead to improved rice yields [11,28,32,65].

In general, few studies have reported any potential negative effects of soil silicon on seedling development. However, in several of our experiments, where we weighed or measured control seedlings without exposure to biotic stresses, we found that seedling growth rates declined with increasing soil silicon concentrations. This was apparent for IR22 (a non-significant trend in Figure 1) and IR50404 (Tables S7 and S8) and suggests that high concentrations of silicon in the soil could slow growth rates as the silicon is assimilated into the plant. These effects might depend on the age of seedlings in the experiments; for example, in experiments using silica gel, and thereby avoiding the confounding effects of any associated nutrients, Horgan et al. (2017) [66] found that silicon reduced the vigor of early seedlings grown in pots; meanwhile, Klotzbücher et al. (2018) [15] found no effect of silicon on rice growth in field plots with older plants. The effects may also relate to inhibition of seedling growth only under very high soil silicon conditions. For example, Hosseini et al. (2012) [64] and Wattanapayapkul et al. (2011) [65] found that seedling growth rates declined at higher soil silicon concentrations (see also IR62 seedlings in Figure 1 from the present study) and, similarly, Annamalai et al. (2022) [58] found that high soil silicon concentrations reduced the germination of rice seed.

Silicon-induced changes to seedling growth rates could affect the tolerance of rice plants to biotic stresses. Tolerance is the capacity of the plant to recover after pest or disease damage. Smaller, slower-growing seedlings are predicted to have lower tolerance but higher resistance against pests and diseases as they assimilate silicon [34,44]. Furthermore, certain aspects of plant growth and anatomy can determine susceptibility to insect herbivores: for example, Hosseini et al., 2012 [64] reported large linear declines in whitehead damage (associated with stripped stemborers: *Chilo suppressalis*) to rice in response to increasing concentrations of furnace slag (a silicon source) in the soil. In the same study, the soil amendments reduced stem diameter, which is a key determinant of resistance to stemborers [41]. Adding nitrogenous fertilizers or other soil nutrients can counter potential silicon-related reductions in seedling growth rates and restore seedling vigor and tolerance [66]. However, nitrogenous fertilizers can also reduce rice resistance against pests and diseases, particularly against phloem-feeding pests, such as planthoppers [43,44,45].

### 4.2. Silicon-Enhanced Resistance against Planthoppers and Leafhoppers

Our results corroborate findings by a number of authors that soil silicon reduces the fitness of BPH nymphs [9,10,63,67,68]. In our experiments, the effects of soil silicon on adult female BPH and egg-laying were generally stronger than the effects on BPH nymphs, and soil silicon was sufficient to reduce BPH egg laying on IR22 to comparable levels with IR62. However, the effects of silicon and resistance were not additive in our experiments, and in many cases, silicon had no apparent effect on levels of resistance in IR62. These results support observations by Salim and Saxena (1992) [69] that silicon reduced feeding, growth, fecundity, and population increases in WBPH on susceptible rice but not on a resistant rice line (*Wbph1*, *Wbph2* genes). Furthermore, He et al. (2015) [68] found that soil silicon had only marginal effects on resistance (*Bph24(t)* gene) to BPH in young, hydroponically-grown rice seedlings. We are unaware of any further comparative studies that have examined the combined effects of silicon and host resistance on planthoppers; it is possible that silicon might have additive effects on some resistant varieties depending on their specific mechanisms of resistance. Such differences in the interactions between soil silicon and resistance will likely be revealed as further resistant varieties are screened for their responses to silicon.

We expected that adding silicon to Vietnamese soils would have a greater effect on locally-adapted planthoppers than similar treatments to Philippine soils. However, this was not the case in our experiments: BPH nymph weights were not affected by soil silicon in our experiments in Vietnam (see also He et al., 2015 [68]), and the response by ovipositing females was more gradual than that observed from experiments conducted in the Philippines (compare Figure 3B and Figure 4D). In Vietnam, soil silicon was associated with low survival of unmated adults in a no-choice bioassay (Figure 4B), but there was no apparent effect of silicon on egg laying in a related no-choice experiment (Figure 4C). In contrast, in choice experiments from the Philippines (Figure 3B) and Vietnam (Figure 4D), silicon clearly reduced BPH egg laying. Such strong antixenosis effects on egg laying, together with reduced longevity in adult females, may explain observations of slower BPH population build-up on rice grown in high-silicon media [10,68]. Although we did not detect soil silicon effects on WBPH (Tables S3 and S6), Salim and Saxena (1992) [69] found silicon amendments strongly influenced WBPH fitness (see above), and Jia et al. (2021) [59] found that soil silicon was associated with reduced nymph settling and egg laying in WBPH as well as lower assimilation and inoculation rates of WBPH-transmitted Southern Rice Black-Streaked Dwarf Virus (SRBSDV).

The relatively strong effects of silicon in our choice experiments (Figure 3) compared to the no-choice experiments (Figure 2) suggests that the mechanisms were largely antixenotic and possibly related to the nature of volatiles emitted from plants grown with added silicon. This further suggests that the silicon was probably associated with enhanced induction of defense responses. For example, Liu et al. (2017) [11] have indicated changes to the volatile blends emitted from rice plants attacked by leaffolders (*Cnaphalocrocis medinalis*) when grown with added silicon. These changes to volatile blends increased the attraction of two parasitoids to rice that was damaged by the leaffolders. Other changes to induced responses or changes to the rates of these responses could affect nymph and adult survival; for example, soil silicon has been associated with increased rates of callose synthesis and changes to the expression of several defense-related genes [9,61,63,67,70]. Higher feeding rates by adult BPH compared to nymphs can result in a larger impact of such rice defenses [71] and might explain why adults were affected by high silicon more than nymphs in our experiments.

### 4.3. Combined Effects of Silicon and Nitrogen on Resistance to Planthoppers and Leafhoppers

Despite wide knowledge of the role of nitrogenous fertilizers in accelerating outbreaks of BPH and in reducing the efficacy of resistant rice varieties against the pest [34,44,45], only Wu et al. (2017) [63] have previously examined the potential for soil silicon amendments to counter these effects. In their study, soil silicon was associated with the reduced expression of genes involved in nitrogen uptake by rice seedlings [63]. At low concentrations, soil silicon amendments (and associated higher plant silicon contents) reduce the concentrations of nitrogen in rice plant tissues and increase C:N ratios [72]. However, at high nitrogen levels, silicon accumulation in rice plant tissues may also decline [63]. We predicted that the effects of high nitrogen in reducing the resistance of IR62 against BPH or in increasing the susceptibility of IR22 would be countered under the influence of soil silicon, particularly at higher soil silicon concentrations. We did not examine potential silicon effects on nitrogen-induced tolerance against the planthoppers. Nitrogen has been shown to enhance rice tolerance against herbivore damage [73], but the effect is only apparent for BPH, where the rice host has at least moderate resistance [44].

In our experiments, resistance reduced the fitness of BPH nymphs (Figure 2, Figure 3), WBPH nymphs (Tables S3 and S6), and GLH nymphs (Table S4). High nitrogen increased the survival and weight of WBPH (Tables S3 and S6) and GLH (Table S4) nymphs and increased the weight of BPH nymphs (Figure 2B) on both IR22 and IR62. These effects of added nitrogen were not countered by increasing soil silicon levels. Silicon was associated with declining BPH nymph survival regardless of nitrogen levels (Figure 2A); but overall, the effects were weak. Meanwhile, in a choice experiment, silicon effectively reduced BPH nymph settling on both IR22 and IR62, but the effect was only apparent under low nitrogen conditions (Figure 3A), suggesting that silicon was not sufficient to reduce preferential settling on high nitrogen plants—or that high nitrogen had reduced the assimilation of silicon into plant tissues. In a further no-choice experiment, we found that high nitrogen had no apparent effects on egg laying by BPH (Figure 2C); however, in the corresponding choice experiment, more eggs were laid in IR62 plants under high nitrogen, but only in the absence of added silicon (Figure 3B). Wu et al. (2017) [63] found that silicon-induced mortality of adult female BPH actually increased under increasing nitrogen levels, despite higher feeding rates under the same nitrogen conditions. When taken together, these results suggest that high levels of nitrogenous fertilizers will diminish the negative effects of both soil silicon and host resistance on the fitness of planthopper and leafhopper nymphs; but that silicon counters nitrogen-related reductions in rice antixenosis defenses, and possibly antibiosis defenses [63], against gravid females.

### 4.4. Effects of Soil Silicon on Disease Resistance

Our results with blast disease suggest that silicon will reduce disease severity; however, the effects were most apparent where plants were moderately susceptible to the inoculations. Plants with effective resistance (IRBL9-W, IRBLz5-Ca, and IRBLz-Fu) and a highly susceptible variety (LTH) were hardly affected by the added silicon (Figure 5). Similarly, silicon consistently slowed lesion development where plants were inoculated with the PXO99 strain of bacterial blight, but the effects were less apparent where plants were inoculated with the less virulent PXO145 strain (Figure 6). Silicon-associated resistance to pathogens has been attributed to the activation of several defense responses, including increased peroxidase and chitinase activities, higher concentrations of soluble phenols [74], and a more efficient stimulation of the terpenoid pathway [75].

PXO99 was clearly a more damaging strain of bacterial blight, as was apparent by the lower weights of PXO99-infected IR24 plants despite lesions of PXO99 and PXO145 having similar lengths in the experiment. Silicon had its greatest effects on final plant dry weights where plants were resistant to bacterial blight (i.e., BB4/PXO99, and BB4, BB7 and IR22/PXO145). Because plant weights increased under increasing soil silicon, but lesion lengths and disease severity were largely unaffected by silicon, we suggest that the effects of silicon included an increase in disease tolerance in the resistant plants. To verify this effect and to compare levels of tolerance under varying amounts of soil silicon, future experiments could compare infected and control (non-infected) plants.

Our results corroborate previous studies that showed soil silicon to reduce the severity of blast diseases and bacterial blight in rice [21,32,76,77,78,79,80]. Applications of silicon nanoparticles to foliage have also been shown to effectively reduce (by 70%) blast disease in rice [81]. In our experiments, the effect of soil silicon in reducing the severity of both diseases was weak relative to the strong effects of the corresponding resistance genes. Previous studies have suggested that soil silicon could reduce the severity of blast in susceptible rice varieties to levels that are comparable with resistant varieties [32]. This effect may be more apparent under field conditions (but see Han et al., 2018 [30]) since several comparative studies conducted under controlled conditions have reported only moderate effects on both susceptible and resistant rice lines [77,82]. Such discrepancies in results may also relate to the ages at which plants are assessed for disease severity. Most laboratory studies are conducted with rice seedlings that are less than 40 days old. Our results indicated a marked plant age effect on the severity of bacterial blight: disease lesions evaluated at 32 days were largely unaffected by soil silicon, but at 59 days, the effects became increasingly apparent (Figure 6). Similarly, Klotzbücher et al. (2018) [15] found no effect of soil silicon on blast disease severity in field plots at 34 days but found a significant linear decline in blast severity with increasing silicon when the plots were resampled at 74 days. The more obvious effects in older, field-grown plants are probably due to a greater accumulation of silicon in plant tissues as they age [77].

### 4.5. Silicon Improves General Rice Plant Health

Our observation of reduced fitness in planthoppers adds to observations of soil silicon effects across a range of rice pests (e.g., *Tibraca limbativentris* [60]; leaffolders [62,72,83,84]; stemborers [33,39,40,64,85]; rice water weevil—*Lissorhoptrus oryzophilus* [28]; and golden apple snail—*Pomacea canaliculata* [66]). Although research has mainly been conducted under controlled conditions, a few studies indicate that these effects can also be observed in rice fields [15,28,30,86]. Furthermore, in our experiments, although the effects of soil silicon were small compared to the relatively large effects of host resistance in reducing the severity of blast and bacterial blight; nevertheless, soil silicon added to the resistance, particularly against the more virulent strain of bacterial blight, and thereby improved seedling growth. Similar effects of silicon in reducing disease severity have been documented across a range of rice pathogens [8,15,21,22,32,79,80,81,82,87].

Our results with planthoppers, blast, and bacterial blight indicate that host resistance remains an important component in reducing pest and disease damage to rice seedlings. However, in contrast to host plant resistance, indications are that silicon can play a significant role in reducing the severity of many disease organisms at once, without the need for targeted resistance genes [15,21,32,65,73,74,78,87,88,89]. Targeted resistance, associated with identified resistance genes, was more effective in protecting rice seedlings in our experiments; however, resistance to such a range of pests and pathogens is not generally common to any single rice variety, and pyramiding resistance genes can result in fitness trade-offs in rice, including yield penalties [90,91]. In contrast, although growth rates may initially decline, adding optimal levels of soil silicon ultimately improves growth and generally enhances seedling quality despite exposure to different biotic stresses, as was observed in our experiments with IR22. Therefore, adding soil silicon to rice paddy fields can increase the overall health of rice plants and may reduce pest and disease incidences at field scales.

## 5. Conclusions

Our results show how silicon-based soil amendments can reduce damage from a range of biotic stresses and thereby increase the general health of rice seedlings. Silicon, assimilated by the rice seedlings, reduced some aspects of planthopper fitness on the susceptible rice variety IR22 to the same levels as observed on the highly resistant variety IR62. However, the effects of soil silicon on BPH were not generally robust against nitrogenous fertilizers. Soil silicon was also associated with a decline in the severity of disease symptoms associated with blast disease and bacterial blight. This reduction in disease severity was influenced by the virulence of the disease strain and the resistance levels of the host plants. Soil silicon stimulated seedling growth in some experiments; however, at high concentrations (4 t SiO_2_ ha^−1^), growth rates often declined. Ultimately silicon soil amendments can directly and indirectly (by reducing biotic stresses) increase seedling weights. Therefore, silicon can be considered a useful component of wider integrated pest management strategies.

## Data Availability

The data presented in this study are available on reasonable request from the corresponding author.

## Supplementary Materials

The following supporting information can be downloaded at: https://www.mdpi.com/article/10.3390/insects13070604/s1, Table S1: Details of experimental set-ups associated with the study; Table S2: Results from no-choice experiments conducted with BPH in the Philippines; Table S3: Results from no-choice experiments conducted with WBPH in the Philippines; Table S4: Results from choice and no-choice experiments conducted with GLH in the Philippines; Table S5: Results from choice experiments conducted with BPH in the Philippines: Table S6: Results from choice experiments conducted with WBPH in the Philippines; Table S7: Results from no-choice experiments conducted with BPH in Vietnam; Table S8: Results from choice experiments conducted with BPH in Vietnam; Table S9: Results from experiments with blast disease conducted in the Philippines; Table S10: Results from experiments with bacterial blight disease conducted in the Philippines; Table S11: Results of repeated measures ANOVA for the bacterial blight experiment.
