# Supplementary material for: Combined Effects of Soil Silicon and Host Plant Resistance on Planthoppers, Blast and Bacterial Blight in Tropical Rice
Source: Insects. 2022 Jul 1;13(7):604. doi: 10.3390/insects13070604 (PMC9318006; doi:10.3390/insects13070604)
Supplement: Supplementary file 1 [file insects-13-00604-s001.zip › insects-1776270-supplementary.pdf]

Supplementary Information

Combined Effects of Soil Silicon and Host Plant Resistance on Planthoppers, Blast and Bacterial Blight in Tropical Rice

Quynh Vu[1,2,3], Gerbert Sylvestre Dossa[3], Enrique A. Mundaca[4], Josef Settele[2,5,6], Eduardo Crisol-Martínez[4,7,8], Finbarr G. Horgan[4,7,9] *

[1] Cuulong Delta Rice Research Institute, Tan Thanh, Thoi Lai District, Can Tho 905660, Vietnam

[2] Helmholtz Centre for Environmental Research – UFZ, Theodor-Lieser-Str. 4, 06120 Halle, Germany

[3] International Rice Research Institute, Makati 1226, Manila, Philippines

[4] Escuela de Agronomía, Facultad de Ciencias Agrarias y Forestales, Universidad Católica del Maule, Casilla 7-D, Curicó 3349001, Chile

[5] iDiv – German Centre for Integrative Biodiversity Research, Puschstrasse 4, 04103, Leipzig, Germany

[6] Institute of Biological Sciences, University of the Philippines (UPLB), 4031 Los Baños, Laguna, Philippines

[7] EcoLaVerna Integral Restoration Ecology, Bridestown, Kildinan, County Cork, T56 P499, Ireland

[8] Association of Fruit and Vegetable Growers of Almeria (COEXPHAL), Carretera de Ronda 11, 04004 Almeria, Spain

[9] Centre for Pesticide Suicide Prevention, University/BHF Centre for Cardiovascular Science, University of Edinburgh, Edinburgh EH16 4TJ, UK

Table S1. Details of experimental set-ups associated with the study.

Silicon Levels (t ha^{-1})	Nitrogen Levels (Kg ha^{-1})	Rice Varieties[1]	Location	Pest or Pathogen[2]	Life Stage/ Pathotype	Bioassay	Plant Age at First Exposure to Silicon[3]	Plant Age at Infestation[3]	Plant Age at Evaluation[3]	N[4]	Total Number of Pots or Arenas
0, 0.25, 1, 4	0, 150	IR22 (S), IR62 (R)	Philippines	BPH, WBPH, GLH	Nymph	Survival and weight gain	0 DAS	20 DAS	35 DAS	6	288
0, 0.25, 1, 4	0, 150	IR22 (S), IR62 (R)	Philippines	BPH, WBPH, GLH	Nymph	Settling (choice)	0 DAS	20 DAS	25 DAS	6	18
0, 0.25, 1, 4	0, 150	IR22 (S), IR62 (R)	Philippines	BPH, WBPH	Adult female	Oviposition	0 DAS	20 DAS	23 DAS	6	192
0, 0.25, 1, 4	0, 150	IR22 (S), IR62 (R)	Philippines	BPH, WBPH	Nymph	Oviposition (choice)	0 DAS	20 DAS	23 DAS	6	12
0, 0.25, 1, 4	0	IR50404 (S)	Vietnam	BPH	Nymph	Survival and weight gain	0 DAS	20 DAS	35 DAS	6	24
0, 0.25, 1, 4	0	IR50404 (S)	Vietnam	BPH	Adult female (unmated)	Survival	0 DAS	30 DAS	35 DAS	6	24
0, 0.25, 1, 4	0	IR50404 (S)	Vietnam	BPH	Adult female (mated)	Oviposition	0 DAS	20 DAS	23 DAS	6	24
0, 0.25, 1, 4	0	IR50404 (S)	Vietnam	BPH	Adult female (mated)	Oviposition (choice)	0 DAS	20 DAS	23 DAS	6	6
0, 0.25, 1, 4	0	IR22 (S), IR24 (S), CO39 (S), LTH (S), IRBL9-W (R), IRBLz5-Ca (R), IRBLz-Fu (R)	Philippines	Blast	M39-1-3-8-1	Disease severity	0 DAS	14 DAS	21 DAS	3	84
0, 0.25, 1, 4	0	IR22 (S), IR24 (S), BB4 (R), BB7 (R), BB67 (R)	Philippines	Bacterial blight	PXO99, PXO145	Disease severity and plant weight	0 DAS	21, 45 DAS	32 DAS, 59 DAS	6	240
0, 0.25, 1, 4	0, 150	IR22 (S), IR62 (R)	Philippines			Growth rates	5 DAS	NA	35 DAT	6	96

[1]: R = resistant, S = susceptible; [2]: BPH = Brown Planthopper, WBPH = Whitebacked Planthopper, GLH = Green Leafhopper; [3]: DAS = Days after Sowing; DAT = Days after transplanting; NA = Not applicable; [4]: N = number of replicates

Table S2. Results from no-choice experiments conducted with brown planthopper in the Philippines. Numbers are means ± SEM.

Variety	Nitrogen Level (Kg ha^{-1})	Silicon Level (t ha^{-1})	Survival (Proportion)	2nd Instars (Proportion)	3rd Instars (Proportion)	4th Instars (Proportion)	5th Instars (Proportion)	Development to Adult (Proportion)	Nymph Weight (mg)	Nymph DW per Plant DW[2] (mg g^{-1})	Number of Eggs per Plant	Eggs per g Plant DW[2]
IR22	0	0.00	0.92±0.03	0.00±0.00	0.00±0.00	0.00±0.00	0.04±0.04	0.96±0.04	9.43±1.18	16.48±2.54	171.67±21.24	218.68±45.60
		0.25	0.80±0.07	0.00±0.00	0.00±0.00	0.00±0.00	0.04±0.04	0.96±0.04	6.70±1.18	12.71±3.81	94.67±9.28	118.54±23.08
		1.00	0.82±0.05	0.00±0.00	0.00±0.00	0.00±0.00	0.00±0.00	1.00±0.00	8.66±0.82	17.87±2.97	150.33±18.81	189.81±32.84
		4.00	0.82±0.07	0.00±0.00	0.00±0.00	0.00±0.00	0.07±0.07	0.93±0.07	7.97±0.78	19.06±4.86	89.17±17.53	122.57±34.88
	150	0.00	0.92±0.03	0.00±0.00	0.00±0.00	0.00±0.00	0.00±0.00	1.00±0.00	11.59±0.60	8.73±0.96	185.00±12.11	85.18±16.24
		0.25	0.85±0.02	0.00±0.00	0.00±0.00	0.00±0.00	0.06±0.04	0.94±0.04	10.00±0.87	7.29±1.24	106.67±13.88	55.92±12.77
		1.00	0.85±0.06	0.00±0.00	0.00±0.00	0.00±0.00	0.07±0.05	0.93±0.05	11.00±1.56	8.44±1.46	128.83±13.99	56.66±6.12
		4.00	0.92±0.05	0.00±0.00	0.00±0.00	0.00±0.00	0.06±0.06	0.94±0.06	9.69±0.83	7.62±0.83	117.50±17.50	58.11±8.15
IR62	0	0.00	0.87±0.06	0.00±0.00	0.12±0.05	0.23±0.06	0.32±0.06	0.34±0.15	3.65±0.66	7.25±1.79	72.00±8.56	76.77±14.13
		0.25	0.65±0.10	0.00±0.00	0.03±0.03	0.15±0.10	0.35±0.16	0.48±0.21	2.57±0.75	3.75±0.74	62.83±8.66	90.70±14.35
		1.00	0.72±0.09	0.03±0.03	0.15±0.10	0.22±0.10	0.30±0.14	0.30±0.15	2.61±0.82	3.29±1.11	87.50±18.53	64.53±12.26
		4.00	0.65±0.07	0.00±0.00	0.05±0.05	0.35±0.12	0.28±0.08	0.32±0.17	2.08±0.36	2.97±0.50	98.67±11.20	122.48±32.01
	150	0.00	0.80±0.05	0.00±0.00	0.00±0.00	0.00±0.00	0.26±0.10	0.74±0.10	4.37±0.75	3.34±0.44	74.17±5.97	34.43±4.80
		0.25	0.62±0.09	0.00±0.00	0.00±0.00	0.04±0.04	0.14±0.10	0.82±0.14	4.80±0.66	5.27±1.05	76.17±7.64	62.41±23.62
		1.00	0.60±0.09	0.00±0.00	0.00±0.00	0.07±0.07	0.11±0.05	0.83±0.10	2.92±1.00	2.60±1.14	78.00±7.90	46.25±5.23
		4.00	0.73±0.07	0.00±0.00	0.00±0.00	0.04±0.04	0.17±0.04	0.80±0.05	4.05±0.55	3.23±0.45	73.50±12.64	37.62±8.51
F-Variety (V)[1]			19.033***					51.236***	185.299***	76.132***	66.329***	21.222***
F-Silicon (S)[1]			3.508*					0.473ns	1.885ns	1.058ns	7.718***	0.920ns
F-Nitrogen (N)[1]			0.015n					5.907*	17.478***	0.340ns	0.063ns	49.946***
S×N[1]			0.380ns					0.134ns	1.382ns	0.889ns	0.904ns	0.804ns
V×N[1]			2.095ns					8.967***	1.473ns	11.448***	0.986ns	7.489**
V×S[1]			1.075ns					1.105ns	0.552ns	1.204ns	8.979***	4.568***
V×SN[1]			0.093ns					0.489ns	0.302ns	0.813ns	1.234ns	2.271ns
Block[1]			ns					ns	ns	3.012*	2.801*	4.068**
Covariate (plant weight)[1]			ns					ns	ns	7.059**	ns	ns
Contrast (S)[1]			(Q)**					ns	ns	ns	(L)*	ns

[1]: ns = $p > 0.05$, * = $p \le 0.05$, ** = $p \le 0.01$, *** = $p \le 0.001$, L = linear contrast, Q = quadratic contrast; [2]: DW = dry weight; degrees of freedom: variety, nitrogen, variety x nitrogen = 1; silicon, variety x silicon, nitrogen x silicon, and variety x nitrogen x silicon interactions = 3; block = 5, covariate = 1; error degrees of freedom = 80, 75 (where 'block' is included) or 74 (where 'block' and 'covariate' are included);

Table S3. Results from no-choice experiments conducted with whitebacked planthopper in the Philippines. Numbers are means ± SEM.

Variety	Nitrogen (kg ha^{-1})	Silicon (t ha^{-1})	Nymph Survival (Proportion)	Nymph Weight (mg DW)[2]	Number of Eggs per Plant	Number of Eggs per g Plant DW[2]
IR22	0	0	0.80±0.06	0.98±0.11	79.33±33.40	127.65±52.20
		0.25	0.67±0.03	0.89±0.21	103.33±30.60	163.54±46.63
		1	0.93±0.07	1.33±0.11	54.00±30.86	73.76±35.40
		4	0.70±0.06	1.14±0.02	26.00±19.86	26.48±16.58
	150	0	0.90±0.06	2.07±0.10	100.67±45.17	61.25±27.29
		0.25	1.00±0.00	2.19±0.38	95.00±5.00	69.95±3.36
		1	0.93±0.07	1.51±0.36	23.33±12.77	16.04±9.81
		4	0.73±0.18	1.34±0.34	73.00±68.39	51.60±44.01
IR62	0	0	0.23±0.19	0.25±0.18	56.67±27.43	57.34±26.63
		0.25	0.37±0.23	0.40±0.21	29.67±15.30	42.40±15.69
		1	0.30±0.25	0.45±0.25	13.67±6.84	12.78±6.40
		4	0.37±0.18	0.20±0.09	30.67±21.46	63.26±54.34
	150	0	0.30±0.12	0.32±0.06	18.00±6.93	11.98±4.48
		0.25	0.43±0.09	0.33±0.11	23.67±9.74	27.94±11.04
		1	0.73±0.18	0.59±0.18	7.67±7.67	5.99±5.99
		4	0.47±0.12	0.39±0.12	56.00±10.00	31.70±10.74
F-variety(V)[1]			39.911***	106.655***	10.205***	7.930**
F-Silicon (S)[1]			1.250ns	0.810ns	2.050ns	2.099ns
F-Nitrogen (N)[1]			4.266*	14.120***	0.012ns	5.061*
V×S[1]			0.718ns	0.660ns	1.169ns	1.983ns
V×N[1]			0.133ns	8.843**	0.366ns	0.813ns
S×N[1]			0.320ns	1.435ns	1.041ns	1.174ns
V×S×N[1]			1.127ns	2.807ns	0.540ns	0.864ns

[1]: ns = $p > 0.05$; * = $p \leq 0.05$; ** = $p \leq 0.01$; *** = $p \leq 0.005$; [2]: DW = dry weight; degrees of freedom: variety, nitrogen, variety x nitrogen = 1; silicon, variety x silicon, nitrogen x silicon, and variety x nitrogen x silicon interactions = 3, error = 80

Table S4. Results from choice and no-choice experiments conducted with green leafhopper in the Philippines. Numbers are means ± SEM.

Variety	Nitrogen (kg ha^{-1})	Silicon (t ha^{-1})	Nymph Survival (Proportion)	Nymph Weight (mg DW) [2]	Nymphs per Plant (Number Settling)	Nymph Settling (Proportion)
IR22	0	0	0.67±0.07	3.41±0.29	10.67±1.31	0.10±0.01
		0.25	0.70±0.10	3.80±0.91	11.33±1.48	0.10±0.01
		1	0.77±0.09	2.87±0.75	11.67±0.99	0.11±0.01
		4	0.67±0.12	3.27±0.41	10.67±0.80	0.10±0.01
	150	0	0.67±0.09	5.46±0.64	10.50±2.36	0.09±0.02
		0.25	0.87±0.07	5.73±0.61	16.33±1.78	0.15±0.01
		1	0.83±0.09	5.30±0.86	15.00±2.44	0.13±0.02
		4	0.93±0.03	6.03±0.27	8.67±3.01	0.08±0.03
IR62	0	0	0.03±0.03	0.26±0.26	0.83±0.40	0.01±0.00
		0.25	0.07±0.07	0.09±0.05	2.50±0.89	0.02±0.01
		1	0.07±0.03	0.09±0.03	2.17±0.98	0.02±0.01
		4	0.07±0.07	0.05±0.03	1.33±0.95	0.01±0.01
	150	0	0.43±0.24	1.19±0.50	0.83±0.31	0.01±0.00
		0.25	0.03±0.03	0.23±0.13	0.67±0.33	0.01±0.00
		1	0.03±0.03	0.05±0.03	2.50±1.15	0.02±0.01
		4	0.07±0.03	0.05±0.03	4.50±1.48	0.04±0.01
F-Variety (V)[1]			241.231***	319.145***	176.680***	209.832***
F-Silicon (S)[1]			1.254ns	0.833ns	1.976ns	0.940ns
F-Nitrogen (N)[1]			6.701**	28.927***	1.715ns	0.201ns
V×S[1]			0.752ns	0.883ns	2.486ns	3.246*
V×N[1]			0.547ns	18.513***	0.566ns	0.007ns
S×N[1]			1.254ns	0.176ns	0.355ns	0.178ns
V×S×N[1]			1.983ns	0.595ns	2.883*	4.956***

[1]: ns = $p > 0.05$; * = $p \leq 0.05$; ** = $p \leq 0.01$; *** = $p \leq 0.005$; [2]: DW = dry weight; degrees of freedom: variety, nitrogen, variety x nitrogen = 1; silicon, variety x silicon, nitrogen x silicon, and variety x nitrogen x silicon interactions = 3, error = 80

Table S5. Results from choice experiments conducted with brown planthopper in the Philippines. Numbers are means ± SEM.

Variety	Nitrogen (kg ha^{-1})	Silicon (Kg ha^{-1})	Nymphs per Plant (Number Settling)	Nymph Settling (Proportion)	Infested Plant Biomass (g DW) [2]	Eggs per Plant	Eggs per Plant (Proportion)
IR22	0	0	14.50±1.89	0.14±0.02	0.05±0.00	168.00±22.57	0.12±0.02
		0.25	8.67±1.91	0.07±0.01	0.04±0.00	48.50±4.65	0.03±0.00
		1	8.83±3.15	0.08±0.03	0.02±0.00	42.83±11.12	0.03±0.01
		4	7.17±1.94	0.06±0.01	0.04±0.01	112.50±26.92	0.08±0.02
	150	0	10.33±2.60	0.10±0.03	0.07±0.01	217.17±24.14	0.15±0.02
		0.25	9.83±1.85	0.09±0.01	0.04±0.01	50.00±11.89	0.03±0.01
		1	7.17±2.74	0.06±0.02	0.03±0.01	65.67±10.24	0.04±0.01
		4	8.50±1.50	0.07±0.01	0.03±0.00	71.50±23.31	0.05±0.01
IR62	0	0	4.17±1.17	0.04±0.01	0.08±0.00	90.17±38.18	0.06±0.03
		0.25	3.67±1.56	0.03±0.01	0.05±0.00	59.33±21.83	0.04±0.02
		1	1.83±1.14	0.01±0.01	0.05±0.00	63.50±16.51	0.04±0.01
		4	1.00±0.63	0.01±0.00	0.07±0.00	72.00±24.26	0.05±0.02
	150	0	7.33±1.58	0.06±0.01	0.09±0.01	219.33±91.45	0.14±0.04
		0.25	8.17±0.79	0.08±0.01	0.08±0.02	61.83±12.71	0.04±0.01
		1	4.17±0.54	0.04±0.00	0.07±0.00	60.83±13.68	0.04±0.01
		4	7.00±1.15	0.06±0.01	0.07±0.00	77.50±21.02	0.05±0.01
F-variety (V)[1]			9.739***		21.547***	0.010ns	0.111ns
F-Silicon (S)[1]			10.722***		42.819***	10.513***	11.041***
F-Nitrogen (N)[1]			5.073*		11.107***	2.217ns	1.782ns
V×S[1]			0.309ns		13.232***	1.701ns	1.644ns
V×N[1]			0.498ns		0.694ns	0.143ns	0.148ns
S×N[1]			4.266**		6.183***	1.227ns	1.409ns
V×S×N[1]			0.217ns		2.713*	0.607ns	0.700ns
Contrast (S)[1]			(L)***		(L)***	(L)***	(L)***

[1]: ns = $p > 0.05$, * = $p \leq 0.05$, ** = $p \leq 0.01$, *** = $p \leq 0.005$, L = linear contrast; [2]: DW = dry weight; ; degrees of freedom: variety, nitrogen, variety x nitrogen = 1; silicon, variety x silicon, nitrogen x silicon, and variety x nitrogen x silicon interactions = 3, error = 80

Table S6. Results from choice experiments conducted with whitebacked planthopper in the Philippines. Numbers are means ± SEM.

Variety	Nitrogen (kg ha^{-1})	Silicon (Kg ha^{-1})	Nymphs per Plant (Number Settling)	Nymph Settling (Proportion)	Eggs per Plant	Eggs per plant (Proportion)
IR22	0	0	5.17±0.65	0.06±0.01	85.00±43.11	0.10±0.05
		0.25	7.33±1.28	0.08±0.02	16.33±9.49	0.06±0.05
		1	6.38±1.73	0.07±0.02	64.67±53.12	0.07±0.06
		4	8.67±2.08	0.09±0.02	13.33±13.33	0.01±0.01
	150	0	8.67±1.61	0.10±0.02	31.67±24.55	0.04±0.03
		0.25	15.33±3.70	0.17±0.04	120.00±75.06	0.19±0.08
		1	11.33±3.24	0.13±0.04	66.00±6.66	0.14±0.06
		4	7.67±1.09	0.08±0.01	58.33±8.82	0.12±0.05
IR62	0	0	2.33±0.80	0.03±0.01	3.33±3.33	0.00±0.00
		0.25	0.67±0.33	0.01±0.00	16.67±12.02	0.02±0.01
		1	0.67±0.33	0.01±0.00	11.00±7.37	0.02±0.01
		4	1.33±0.49	0.02±0.01	10.00±10.00	0.01±0.01
	150	0	4.17±0.83	0.05±0.01	55.00±35.00	0.06±0.04
		0.25	4.83±1.01	0.06±0.01	61.67±41.77	0.11±0.05
		1	3.50±0.85	0.04±0.01	18.33±11.67	0.02±0.01
		4	2.67±0.33	0.03±0.01	15.00±15.00	0.02±0.02
F-variety(V) [1]				133.239***		9.606***
F-Silicon(S) [1]				0.928ns		1.079ns
F-Nitrogen(N)[1]				29.043***		8.908***
V×S[1]				0.888ns		0.334ns
V×N[1]				2.452ns		0.247ns
S×N[1]				1.371ns		1.014ns
V×S×N[1]				0.299ns		2.486ns

[1]: ns = $p > 0.05$, *** = $p \leq 0.005$; degrees of freedom: variety, nitrogen, variety x nitrogen = 1; silicon, variety x silicon, nitrogen x silicon, and variety x nitrogen x silicon interactions = 3, error = 80

Table S7. Results from no-choice experiments conducted with brown planthopper in Vietnam. Numbers are means ± SEM.

Silicon (t ha^{-1})	4th Instars (Proportion)	5th Instars (Proportion)	Development to Adult (Proportion)	Females (Proportion)	Nymph Survival (Proportion)	Nymph Weight (mg DW) [2]	Infested Plant Height (cm)	Infested Plant Weight (g DW) [2]	Control Plant Height (cm)	Control Plant Weight (g DW) [2]	Nymph Weight per Plant (mg g-DW^{-1}) [2]	Eggs per Plant	Eggs Inserted to Midrib (Proportion)	Adult Survival (Proportion)
0	0.04±0.03	0.43±0.09	0.53±0.10	0.44±0.13	0.68±0.14	2.06±0.57	49.33±2.01	0.73±0.07	52.33±2.55	0.90±0.10	2.87±0.72	30.50±11.92	0.39±0.14	0.67±0.21
0.25	0.09±0.04	0.63±0.10	0.28±0.10	0.29±0.14	0.43±0.08	1.23±0.25	52.67±2.30	1.00±0.15	51.83±3.15	0.97±0.19	1.47±0.39	89.83±29.37	0.18±0.08	1.00±0.00
1	0.09±0.04	0.53±0.12	0.37±0.14	0.56±0.12	0.62±0.06	1.93±0.24	54.83±1.85	1.05±0.08	51.33±2.96	0.89±0.18	1.89±0.31	31.33±13.50	0.16±0.06	0.50±0.22
4	0.03±0.03	0.51±0.06	0.47±0.07	0.54±0.10	0.57±0.10	1.80±0.32	51.67±2.70	0.92±0.15	50.33±2.30	0.88±0.23	2.07±0.49	50.50±12.96	0.37±0.10	0.00±0.00
F-Silicon[1] Contrast[1]			1.211ns	0.693ns	1.136ns	0.976ns	1.037ns	1.452ns	0.096ns	0.125ns	1.367ns	2.277ns	0.896ns	5.901*** (L)**

[1]: ns = $p > 0.05$, ** = $p \leq 0.01$, *** = $p \leq 0.005$, L = linear contrast; [2]: DW = dry weight; degrees of freedom: silicon = 3, error = 20

Table S8. Results from choice experiments conducted with brown planthopper in Vietnam. Numbers are means ± SEM.

Silicon (t ha^{-1})	Eggs per Plant (Proportion)	Plant Weight (g DW)[2]	Eggs per g Plant DW[2]
0	0.41±0.04	0.49±0.13	256.77±68.43
0.25	0.28±0.04	0.49±0.12	170.61±68.43
1	0.17±0.02	0.42±0.06	121.01±68.43
4	0.14±0.07	0.36±0.03	70.90±68.43
F-Silicon[1]	13.333***	0.442ns	7.967***
Contrast[1]	(L)***		(L)****

[1]: ns = $p > 0.05$, ** = $p \leq 0.01$, *** = $p \leq 0.005$, L = linear contrast; [2]: DW = dry weight; degrees of freedom: silicon = 3, error = 20

Table S9. Results from experiments with blast disease conducted in the Philippines. Numbers are means ± SEM.

Rice Genotype	Silicon (t ha^{-1})	SES Score[2]
CO39	0	4.33±0.33dB
CO39	0.25	4.33±0.33AB
CO39	1	4.33±0.33A
CO39	4	3.67±0.33A
LTH	0	4.67±0.33d
LTH	0.25	5.00±0.00
LTH	1	5.00±0.00
LTH	4	4.67±0.33
IR22	0	4.00±0.00c
IR22	0.25	2.33±0.33
IR22	1	2.67±0.33
IR22	4	2.67±0.33
IR24	0	4.00±0.00c
IR24	0.25	3.00±0.58
IR24	1	3.00±0.58
IR24	4	2.67±0.67
IRBLZ5-Ca	0	2.00±0.00b
IRBLZ5-Ca	0.25	2.00±0.00
IRBLZ5-Ca	1	1.00±0.58
IRBLZ5-Ca	4	1.67±0.33
IRBL9-W	0	1.00±0.00a
IRBL9-W	0.25	1.00±0.00
IRBL9-W	1	0.33±0.33
IRBL9-W	4	0.67±0.33
IRBLZ-Fu	0	0.67±0.33a
IRBLZ-Fu	0.25	0.67±0.33
IRBLZ-Fu	1	0.00±0.00
IRBLZ-Fu	4	0.67±0.33
F-Silicon (S)[1]		5.048***
F-Genotype (G) [1]		99.369***
S×G[1]		0.451ns
Contrast (S)[1]		(L)***

[1]: ns = $p > 0.05$, *** = $p > 0.001$, L = linear contrast; [2]: SES = Standard Evaluation System, uppercase letters indicate homogenous silicon groups, lowercase letters indicate homogenous variety groups; degrees of freedom: genotype = 6, silicon = 3, interaction = 18, error = 56

Table S10. Results from experiments with bacterial blight disease conducted in the Philippines. Numbers are means ± SEM.

Genotypes	Silicon (t ha^{-1})	PXO99 Lesion Length (mm) at 32 DAS[1]	PXO99 Plant Weight (g DW) at 32 DAS[1]	PXO145 Lesion Length (mm) at 32 DAS[1]	PXO145 Plant Weight (g DW) at 32 DAS[1]	PXO99 Lesion Length (mm) at 59	PXO99 Plant Weight (g DW) at 59 DAS[1]	PXO145 Lesion Length (mm) at 59 DAS[1]	PXO145 Plant Weight (g DW) at 59 DAS[1]
BB4	0	7.86±1.87	0.74±0.11	1.79±0.68	1.34±0.06	19.30±3.29	6.40±0.92	3.48±0.67	11.99±1.43
	0.25	5.31±0.80	0.96±0.15	1.24±0.22	1.16±0.18	8.56±0.88	8.88±0.89	2.93±0.56	10.61±0.77
	1	6.39±1.07	1.13±0.08	1.77±0.17	1.56±0.09	9.16±1.26	9.09±0.93	3.04±0.82	11.90±1.13
	4	7.03±1.50	0.97±0.09	3.01±0.71	1.63±0.39	10.97±0.94	9.01±1.47	3.03±0.35	14.21±0.57
BB67	0	9.05±0.81	0.60±0.05	0.86±0.22	1.36±0.20	20.82±2.24	7.27±0.97	1.64±0.27	9.45±0.96
	0.25	10.94±1.06	0.63±0.08	1.05±0.12	1.17±0.10	18.90±2.16	6.71±1.05	1.58±0.27	10.31±1.34
	1	10.02±1.52	0.95±0.14	1.37±0.27	1.48±0.19	15.51±2.34	9.03±0.97	4.61±2.10	10.19±1.59
	4	9.04±0.40	0.70±0.11	0.68±0.19	1.14±0.16	23.65±3.11	5.95±0.67	5.84±2.62	9.96±1.05
BB7	0	9.97±1.36	0.93±0.17	3.33±1.02	1.42±0.22	30.93±1.26	4.05±0.70	5.69±0.70	10.02±0.71
	0.25	8.18±1.48	0.64±0.05	3.11±1.05	1.22±0.12	24.79±3.07	5.53±0.82	6.95±2.95	9.84±1.19
	1	9.93±1.28	0.89±0.19	3.99±1.41	1.52±0.19	29.10±2.09	4.70±0.82	4.94±0.80	9.81±1.12
	4	10.16±0.95	0.79±0.13	3.83±1.04	1.62±0.30	26.35±2.17	6.95±0.95	5.63±1.19	13.11±1.50
IR22	0	9.20±1.73	0.63±0.08	1.54±0.33	1.39±0.07	31.21±1.59	5.18±0.91	10.47±1.62	11.72±0.93
	0.25	9.69±1.31	0.79±0.17	2.32±0.28	1.20±0.07	25.06±2.49	2.73±0.39	10.23±0.80	8.68±1.82
	1	9.68±1.07	0.87±0.10	2.29±0.17	1.72±0.19	25.12±3.52	3.61±0.38	9.09±1.97	11.89±1.08
	4	9.30±0.94	1.06±0.04	2.53±0.40	1.84±0.21	24.58±2.17	4.37±0.44	9.80±1.61	12.49±1.18
IR24	0	10.26±0.53	0.91±0.13	8.26±1.18	1.24±0.15	29.74±1.99	5.19±0.66	20.75±1.76	12.88±1.44
	0.25	11.27±1.60	1.20±0.16	10.41±1.12	1.35±0.22	30.30±1.25	4.42±0.45	25.21±1.55	11.27±1.02
	1	9.24±0.96	0.88±0.16	9.97±0.98	1.23±0.20	29.08±3.18	6.62±0.85	25.49±1.39	12.21±1.01
	4	9.06±1.05	0.93±0.20	9.30±0.61	1.17±0.20	27.55±3.11	4.40±0.84	25.57±0.77	11.22±1.26

[1]: DAS = days after sowing; DW = dry weight

Table S11. Results of repeated measures ANOVA for the bacterial blight experiment (see means in Table S10).

Within-Subject Effects	DF	F-Lesion	P-Value	F-Weight	P-Value
Plant age	1	747.102	0.001	2030.165	0.001
Plant age × BB type	1	133.308	0.001	205.681	0.001
Plant age × genotype	4	44.361	0.001	8.373	0.001
Plant age × silicon	3	1.940	0.124	2.254	0.083
Plant age × BB type × genotype	4	8.995	0.001	7.452	0.001
Plant age × BB type × silicon	3	2.858	0.038	1.348	0.260
Plant age × genotype × silicon	12	1.248	0.253	1.652	0.080
Plant age × BB type × genotype × silicon	12	0.457	0.938	0.461	0.935
Error	200				
**Between Subject Effects**					
BB type	1	820.027	0.001	316.031	0.001
Genotype	1	152.053	0.001	8.765	0.001
Silicon	4	1.512	0.212	4.231	0.006
BB type × genotype	3	39.687	0.001	7.676	0.001
BB type × silicon	4	6.014	0.001	1.971	0.120
Genotype × silicon	3	1.910	0.035	2.005	0.025
BB type × genotype × silicon	12	0.937	0.511	0.576	0.860
Error	200				
